# Menstrual disturbances in women with advanced heart failure and heart transplant recipients

**DOI:** 10.3389/fcvm.2025.1466964

**Published:** 2026-01-27

**Authors:** Noelia Fernández Villa, Alba María García García, Miriam Gómez Molina, David José Vázquez Andrés, Francisco José Pastor Pérez, Iris Paula Garrido Bravo, Domingo Andrés Pascual Figal

**Affiliations:** 1 Cardiology Department, Hospital Clínico Universitario Virgen de la Arrixaca, Murcia, Spain; 2 Advanced Heart Failure and Heart Transplant Unit, Hospital Clínico Universitario Virgen de la Arrixaca, Murcia, Spain; 3 Cardiology Department, Hospital Virgen del Castillo, Murcia, Spain

**Keywords:** advanced heart failure, heart transplantation, menorrhagia, menstrual disorders, menstrual disturbances

## Abstract

**Background:**

Menstrual disturbances (MDs) are common among women with advanced heart failure. These disorders can arise from hormonal, hemodynamic, pharmacological, and psychological factors. Heart transplantation (HT) involves changes in the cardiovascular system and requires adjustments in medical treatment. However, the prevalence and impact of MDs before and after HT have not been thoroughly evaluated.

**Methods:**

We conducted a single-center retrospective observational study that included all female heart transplant recipients followed from 1999 to 2020. A questionnaire was used to assess the menstrual bleeding patterns before and after HT.

**Results:**

Data from 19 female heart transplant recipients were collected. The median age at HT was 57 (IQR: 35–60) years. 15 of these women were of childbearing age at heart disease diagnosis, and among them, 9 (60%) experienced MDs before the transplant. At the time of HT, 8 women remained of reproductive age, and all reported MDs during the post-transplant period. The most common event was menorrhagia, affecting 5 of 15 (33.3%) patients of reproductive age before HT and 6 of 8 (75%) women still of childbearing age after HT. In 2 heart transplant recipients, menorrhagia improved after the withdrawal of antiplatelet therapy. Additionally, 2 women developed early menopause, one of them before HT and the other afterwards. No cases of postmenopausal bleeding were reported.

**Conclusion:**

MDs are prevalent in patients with advanced heart failure and those who undergo HT. Changes in hemodynamic status and medical treatment may be associated with these disorders. Further studies are needed to assess these issues.

## 1 Introduction

Menstrual disturbances (MDs) are common among women with advanced heart diseases (1, 2). However, the pathophysiology of these disorders is poorly understood in these patients. Moreover, MDs are also reported after heart transplantation (HT) (1, 3). Heart transplant recipients experience changes in cardiovascular and hormonal status and require modifications in their medical treatment. These factors may be related to the development of MDs in these patients.

To date, research on MDs and their association with heart diseases and solid organ transplantation has been limited, particularly among women who have undergone a heart transplant, and evidence on this topic is scarce. Some reviews have evaluated the presence of MDs in women with congenital heart disease (1, 2). In one of these studies, including 114 young women with congenital heart disease, 83% of participants reported experiencing one or more MDs (1). In another prospective analysis of 304 young women with congenital heart disease, the incidence of MDs was also significant (40% in the complex congenital heart disease group vs. 25% in the simple congenital heart disease group). Notably, menorrhagia was the most frequently reported dysfunction in this study (2). On the other hand, in an observational study conducted by Davis-Kankanamge et al. (3), the prevalence of MDs increased among young women after receiving a solid organ transplant (23.8% of study participants were heart transplant recipients). However, there is a lack of conclusive studies that specifically assess the presence of MDs in women with advanced heart failure or heart transplant recipients.

Therefore, this study aimed to assess the prevalence of MDs in a group of female heart transplant recipients. For this purpose, we evaluated the menstrual cycle characteristics in these patients before and after HT and described possible factors related to these disorders.

## 2 Materials and methods

A single-center, observational, and descriptive study was designed to involve all women over 18 years of age who underwent HT at Virgen de la Arrixaca University Clinical Hospital between 1999 and 2020. Patients who were unable to provide sufficient information about their menstrual pattern or who died during the follow-up period were excluded.

For each participant who met the inclusion criteria, we gathered data on their menstrual bleeding patterns in the pre-transplant period (defined as the time from heart disease diagnosis to HT) and the post-transplant period (after the procedure). This information was collected retrospectively through telephone interviews or clinical visits conducted by cardiologists from the Heart Transplant Unit. For women of childbearing age during either of these periods who reported MDs, the specific MDs were also recorded. For menopausal women at the time of heart disease diagnosis, only postmenopausal bleeding was considered. The different types of MDs were classified according to the criteria outlined in Table 1.

**Table 1:** Definitions of menstrual disturbances.

Menstrual disturbances	Definitions
Dysmenorrhea	Recurrent pelvic or lower abdominal pain during menstruation.
Polymenorrhea	Menstrual cycle shorter than 22 days. More than four bleeding episodes in a 90-day period.
Oligomenorrhea	Menstrual cycle occurring at intervals of more than 35 days. Up to two bleeding episodes in a 90-day period.
Amenorrhea	Absent menstrual bleeding in a woman of reproductive age in a 90-day period.
Menorrhagia	Heavy and prolonged menstrual bleeding (> 80 mL or > 7–8 days).
Metrorrhagia	Dysfunctional uterine bleeding occurring outside the expected menstrual cycle.
Hypomenorrhea	Abnormally light menstrual flow (<30 mL) or short duration of bleeding (<2-3 days).
Premature menopause	Menopause occurs before age 40.
Early menopause	Menopause occurs between the ages of 40 and 45.
Postmenopausal bleeding	Vaginal bleeding that occurs more than 12 months after a woman’s last menstrual period.

Furthermore, we collected other demographics and clinical variables, including age at heart disease diagnosis, etiology of cardiomyopathy, age at HT, use of oral anticoagulation and antiplatelet drugs both before and after HT, and the type of immunosuppressive therapy these patients received following HT. These data were retrospectively obtained through electronic medical records available in Selene® (Corporate Hospital Information System of Murcia Health Institution).

The primary outcome of the study was the occurrence of MDs, analyzed as a dichotomous variable (presence or absence). We evaluated this variable both before and after HT and compared the results obtained for each period. As secondary objectives of the research, we assessed the prevalence of different types of MDs among women of reproductive age in each period, and also described the use of antiplatelet and oral anticoagulant drugs among patients who experienced heavy menstrual bleeding.

Descriptive statistical analysis was performed using SPSS Statistics for Windows, version 25.0 (IBM, Armonk, New York, USA). Categorical variables were expressed as numbers and percentages, while quantitative variables were summarized as means and standard deviations (SD) or medians and interquartile ranges (IQR), as appropriate.

## 3 Results

Between 1999 and 2020, our center performed a total of 173 heart transplants, 45 (26%) of which were in women. During this follow-up period, 23 women were excluded due to mortality. Among the remaining 22 women, we were unable to collect sufficient menstrual pattern information for 3 of them. Consequently, 19 female heart transplant recipients were ultimately included in the study. Demographic and clinical characteristics of the study population are presented in Table 2.

**Table 2:** Demographic and clinical characteristics of the study population.

Case	Etiology of cardiomyopathy	Age at HD diagnosis (years)	Age at HT (years)	Immunosuppressive therapy after HT	Antiplatelet or anticoagulant drugs before HT	Antiplatelet or anticoagulant drugs after HT	MDs in women of reproductive age before HT (*n* = 15)^£^	MDs in women of reproductive age after HT (*n* = 8)^†^
1	NIDCM	Unknown	59^&^	CSA + MMF	No	Clopidogrel	No	–
2	IDCM	Unknown	62^&^	CSA + MMF	ASA	ASA	Yes	–
3	HCM	50	58^&^	CSA + EVR + PD	Acenocoumarol	ASA	Yes	–
4	NIDCM	13	14	TAC + MMF	ASA	No	No	Yes
5	NIDCM	33	42	TAC + MMF	ASA	Acenocoumarol	No	Yes
6	NIDCM	39	42	TAC + EVR	ASA	ASA	Yes	Yes
7	NIDCM	20	21	TAC + MMF + PD	No	Clopidogrel	Yes	Yes
8	HCM	45	68^&^	TAC + MMF	Acenocoumarol	ASA	Yes	–
9	HCM	46	55^&^	TAC + MMF + PD	Acenocoumarol	ASA	No	–
10	NIDCM	39	67^&^	CSA + MMF + PD	Acenocoumarol	No	Yes	–
11	IDCM	56*	57^&^	TAC + MMF + PD	ASA + Clopidogrel + Acenocoumarol	No	–	–
12	NIDCM	55*	60^&^	TAC + EVR + PD	No	ASA	–	–
13	NIDCM	28	35	TAC + MMF + PD	No	ASA	No	Yes
14	HCM	16	22	TAC + MMF + PD	No	ASA	No	Yes
15	IDCM	44	45	TAC + EVR + PD	ASA + Ticagrelor	ASA	Yes	Yes
16	HCM	43	58^&^	TAC + MMF + PD	Acenocoumarol	ASA	Yes	–
17	RCM	22	28	TAC + MMF + PD	Acenocoumarol	No	Yes	Yes
18	HCM	58*	64^&^	CSA + MMF + PD	Acenocoumarol	No	–	–
19	IDCM	56*	57^&^	TAC + MMF + PD	ASA + Ticagrelor	No	–	–
**Total**	–	** 43 (IQR: 25–53)**	** 57 (IQR: 35–60)**	–	**14 (73,7%)**	**13 (68,4%)**	** 9 (60%)**	** 8 (100%)**

ASA, acetylsalicylic acid; CSA, cyclosporine; EVR, everolimus; HCM, hypertrophic cardiomyopathy; HD, heart disease; HT, heart transplantation; IDCM, ischemic dilated cardiomyopathy; MDs, menstrual disturbances; MMF, mycophenolate-mofetil; NIDCM, non-ischemic dilated cardiomyopathy; PD, prednisone; RCM, restrictive cardiomyopathy; TAC, tacrolimus.

Data are expressed as *n* (%) or median (interquartile range).

* Postmenopausal women at heart disease diagnosis (*n* = 4).

& Postmenopausal women at HT (*n* = 11).

£ Refers to patients of reproductive age in the pre-transplant period (*n* = 15).

† Refers to patients still of reproductive age after HT (*n* = 8).

The median age of study patients at heart disease diagnosis was 43 (IQR: 25–53) years, and the median age at the time of HT was 57 (IQR: 35–60) years. Non-ischemic cardiomyopathy was diagnosed in 15 (78.9%) participants, with the remaining 4 (21.1%) having ischemic dilated cardiomyopathy.

Regarding menopausal status, at heart disease diagnosis, 15 (78.9%) women were of reproductive age, whereas 4 (21.1%) had already reached menopause. At the time of HT, 8 (42.1%) of the women were still of childbearing age, while the remaining 11 (57.9%) were menopausal.

In terms of medical treatment, before HT, 7 (36.8%) women received antiplatelet drugs, and 8 (42.1%) women were anticoagulated with acenocoumarol. After HT, 12 (63.2%) patients received antiplatelet treatment, and only 1 (5.3%) was anticoagulated. Tacrolimus was the most commonly used calcineurin inhibitor in immunosuppressive therapy after HT. As for the second immunosuppressant, 4 (21.1%) patients were treated with everolimus, while the remaining 15 (78.9%) subjects received mycophenolate mofetil. Furthermore, 13 (68.4%) patients were taking corticosteroids.

In our population, 9 of 15 (60%) women of reproductive age in the pre-transplant period experienced MDs. Following transplantation, all women who remained of reproductive age (*n* = 8; 100%) developed one or more MDs (Table 2). No cases of postmenopausal bleeding were reported in patients who had reached menopause before or after HT.

Menorrhagia was the most common issue, especially during the post-transplant period. This disorder was reported by 5 of 15 (33.3%) women of reproductive age before HT and by 6 of 8 (75%) women still of childbearing age after the transplant. Among patients who had menorrhagia before HT (*n* = 5), 4 (80%) were receiving antiplatelet or anticoagulant therapy during this period. In contrast, the use of antiplatelet or anticoagulant drugs was lower in reproductive-age women who did not have menorrhagia before HT (*n* = 7; 70%). Among those who developed menorrhagia after HT (*n* = 6), 4 (66.7%) were initially taking antiplatelet drugs, and 2 of these cases improved after discontinuing this treatment. Additionally, 2 (33.3%) patients required intrauterine device implantation to control menstrual bleeding, and 1 (16.7%) patient needed intravenous iron and tranexamic acid administration to treat severe anemia caused by heavy menstrual bleeding (Table 3). The 2 patients of reproductive age who did not develop post-transplant menorrhagia were receiving antithrombotic treatment.

**Table 3:** Management of reproductive-age women who experienced menorrhagia in the post-transplant period.

Case	Initial antiplatelet or anticoagulant therapy	Withdrawal of antithrombotic therapy during follow-up	Other therapies to control menorrhagia
1	No	-	No
2	Yes	No	No
3	Yes	Yes	Intrauterine device implantation
4	Yes	No	Intravenous iron + tranexamic acid
5	Yes	Yes	No
6	No	-	Intrauterine device implantation
**Total**	**4 (66.7%)**	**2 (50%)**	**3 (50%)**

Data are expressed as *n* (%).

The second most common event observed before HT was oligomenorrhea, which occurred in 4 of 15 (26.7%) women of reproductive age in that period. However, no cases were reported after the transplant. In the post-transplant period, the second most frequently reported MDs were transient amenorrhea and dysmenorrhea, each affecting 3 of 8 (37.5%) women who remained of childbearing age after the transplant. Amenorrhea was observed during the first 3–6 months after surgery, and all cases subsequently recovered their normal menstrual cycle. Additionally, 2 women developed early menopause (< 45 years of age), one of them before HT and the other following it. Table 4 summarizes the frequency of the different types of MDs reported in reproductive-age women before and after HT.

**Table 4:** Prevalence of different types of menstrual disturbances before and after heart transplantation among reproductive-age women in each period.

Menstrual disturbances	Prevalence before HT (*n* = 15)	Prevalence after HT (*n* = 8)
Dysmenorrhea	1 (6.7%)	3 (37.5%)
Polymenorrhea	0	1 (12.5%)
Oligomenorrhea	4 (26.7%)	0
Amenorrhea	0	3 (37.5%)
Menorrhagia	5 (33.3%)	6 (75.0%)
Metrorrhagia	0	1 (12.5%)
Hypomenorrhea	1 (6.7%)	0
Premature menopause	0	0
Early menopause	1 (6.7%)	1 (12.5%)
Any menstrual disturbances	9 (60%)	8 (100%)

HT, Heart transplantation.

Data are expressed as *n* (%).

## 4 Discussion

Despite available published information on fertility and contraception in young female solid organ transplant recipients (3–5), MDs are a prevalent and understudied issue in these patients. Previous reviews have shown that MDs are present in up to a quarter of these patients, both before and after transplantation. Dysmenorrhea and menorrhagia are among the most common MDs reported in these studies (3). However, research on the actual prevalence and underlying etiology of menstrual cycle disorders in women with heart diseases remains scarce, especially those who received a heart transplant, and limited data available has been collected from young female patients with congenital heart disease (1, 2). Changes in hemodynamic, endocrine, and hormonal status, as well as the side effects of drugs these patients usually take, have been proposed as predisposing factors for the development of MDs (6–11). Additionally, the frequent use of antiplatelet and anticoagulant therapy among patients with advanced heart failure and heart transplant recipients may also contribute to the heavy menstrual bleeding experienced by many of them (1, 12).

In our population of female heart transplant recipients, nearly two-thirds of reproductive-age women at heart disease diagnosis had MDs before the transplant. Post-transplantation, all women who remained of childbearing age developed these disorders. These findings emphasize the high prevalence of this issue in patients with advanced heart failure, particularly those who undergo a heart transplant.

Menorrhagia was the most frequent dysfunction reported in our study, especially after HT. It is important to note that over 65% of patients who had menorrhagia before or after HT were taking antiplatelet or anticoagulant drugs. Similar to previous research (12, 13), these results suggest that these treatments may increase the likelihood of this menstrual disorder occurring. Nevertheless, more studies with larger populations are needed to establish a causal association between the use of antithrombotic therapy and the development of menorrhagia. In addition, heavy menstrual bleeding could significantly affect the comprehensive management of these patients (13). Indeed, some of our patients who had anemia related to menorrhagia after HT required the withdrawal of antiplatelet therapy or other therapeutic measures, such as intravenous iron infusion or intrauterine device implantation, to control menstrual bleeding, findings that support this hypothesis.

In addition, transient amenorrhea was also a common issue immediately after HT, likely related to the psychological and organic stress that these patients suffered during the perioperative period (6). Moreover, although early menopause was a rare event in our population, both advanced heart failure and HT could also predispose the onset of menopause at a premature age in these patients. However, more studies are needed to confirm the relationship between these two variables.

Thus, our research findings indicate that MDs are common among reproductive-age women with advanced heart failure and female heart transplant recipients. These disorders can impair their quality of life and pose additional challenges for the clinical and therapeutic management of these patients, especially for those who have undergone a heart transplant. Based on our results, we recommend a regular assessment of MDs during the clinical follow-up of these women in Advanced Heart Failure and Heart Transplant Units. Moreover, cardiologists should consider collaborating with other professionals, such as gynecologists and hematologists, to provide optimal care for these patients (13, 14). Additionally, it remains uncertain whether MDs or their effects could affect the medium and long-term prognosis of female heart transplant recipients (7, 15, 16). Further research with long-term follow-up is required to explore this hypothesis.

Regarding the limitations of our study, it is essential to consider its small sample size and single-center retrospective design. There is also a potential for selection bias due to the voluntary nature of participant involvement, as well as recall bias related to the information provided. Additionally, our study population primarily included middle-aged patients, suggesting that the prevalence of MDs may be even higher in younger populations. Despite these limitations, this study is the first to specifically evaluate the prevalence of MDs in patients with advanced heart failure both before and after HT, which represents the main strength of the research.

## 5 Conclusion

MDs are a common and clinically relevant problem in women with advanced heart failure and after HT. Medical treatments and hemodynamic changes related to these clinical conditions may contribute to their development. The impact of MDs on the therapeutic management and prognosis of these patients remains largely unknown. Further research involving larger patient populations is needed to assess these issues.

## Data Availability

The original contributions presented in the study are included in the article/Supplementary Material, further inquiries can be directed to the corresponding author.
